# Computational analyses of drug resistance mutations in *katG* and *emb* complexes in *Mycobacterium tuberculosis*

**DOI:** 10.1002/prot.26684

**Published:** 2024-03-14

**Authors:** Aadam Basrai, Tom L. Blundell, Arun Prasad Pandurangan

**Affiliations:** ^1^ Victor Phillip Dahdaleh Heart and Lung Research Institute, Department of Medicine University of Cambridge Cambridge UK

**Keywords:** antimicrobial drug resistance, ethambutol, infectious diseases, isoniazid, machine learning, molecular docking, *Mycobacterium tuberculosis*, protein stability prediction

## Abstract

The number of antibiotic resistant pathogens is increasing rapidly, and with this comes a substantial socioeconomic cost that threatens much of the world. To alleviate this problem, we must use antibiotics in a more responsible and informed way, further our understanding of the molecular basis of drug resistance, and design new antibiotics. Here, we focus on a key drug‐resistant pathogen, *Mycobacterium tuberculosis*, and computationally analyze trends in drug‐resistant mutations in genes of the proteins *embA*, *embB*, *embC*, and *katG*, which play essential roles in the action of the first‐line drugs ethambutol and isoniazid. We use docking to predict binding modes of isoniazid to *katG* that agree with suggested binding sites found in our laboratory using cryo‐EM. Using mutant stability predictions, we recapitulate the idea that resistance occurs when *katG's* heme cofactor is destabilized rather than due to a decrease in affinity to isoniazid. Conversely, we have identified resistance mutations that affect the affinity of ethambutol more drastically than the affinity of the natural substrate of *embB*. With this, we illustrate that we can distinguish between the two types of drug resistance—cofactor destabilization and drug affinity reduction—suggesting potential uses in the prediction of novel drug‐resistant mutations.

## 1 INTRODUCTION

Antimicrobial resistance (AMR) is quickly becoming one of the biggest challenges we face today, with estimates of 10 million human deaths and economic losses of $100–210 trillion by 2050.^1^ It is for this reason that the WHO has labeled antimicrobial resistance as “One of the biggest threats to global health, food security, and development today,”^2^ indicating that AMR has far reaching socioeconomic consequences. The reasons for an increase in AMR are multi‐faceted, including misuse by patients, oversubscription by doctors, and its large‐scale prophylactic use in the agriculture industry, enabling the spread of resistance through horizontal gene transfer.^3^ AMR will affect the treatment of a variety of pathologies, and decades of research and breakthroughs in diseases such as HIV and malaria. Resistance is also rapidly developing in common pathogens, particularly gram‐negative bacteria which often rapidly acquire drug resistance, and pathogens such as *Pseudomonas aeruginosa* and *Staphylococcus aureus* that are found in hospitals.^4^ It will also severely impact the treatment of immunocompromised patients such as those suffering from cancer or autoimmune diseases, recovering from surgery, or having had an organ transplant. It is evident, therefore, that understanding the root causes of AMR and how these can be alleviated is of utmost importance. Of these, multidrug‐resistant tuberculosis (MDR‐TB) is of significant concern. Globally, an estimated 410 000 people developed MDR or rifampicin resistant TB (RR‐TB) in 2022.^5^ By 2050, MDR‐TB is estimated to have resulted in human losses of $16.7 trillion.^1^ Tuberculosis, caused by *Mycobacterium tuberculosis* (*Mtb*), is typically treated with a strict treatment regimen consisting of four antibiotics, rifampicin and isoniazid (INH) taken for 6 months, and pyrazinamide and ethambutol taken for the first 2 months.^6^ However, resistance to all four of these first‐line drugs is common, with multiple mutations being seen in genes implicated in the use of these drugs, including several hundred in genes such as *rpoB*, *katG*, and *embB* affecting rifampicin, isoniazid, and ethambutol, respectively.^7^ This drug resistance results in the requirement to use second line drugs such as kanamycin and capreomycin and potentially increase treatment to between 9 and 24 months.^6^ While many of these first‐line drugs have been used for over 60 years, their molecular mechanism is still not particularly well understood. In the past several decades, there has been a stagnation in the number of new antibiotics that have come to market, driven by two factors. First, novel antibiotics are difficult to create due to the difficulty in understanding the complex interactions between antibiotics and their targets, resulting in the majority of new antibiotics being simple modifications of existing ones.^8^ Second, there is little economic incentive for the research of novel antibiotics, with an estimate of $1 billion to develop a new antibiotic over 10–15 years, but an average net present value of only $50 million.^3^ The molecular underpinnings of AMR remain poorly understood, in part due to the difficulty in understanding the changes to protein structure from single point mutations, which account for the majority of drug‐resistant mutations. While protein prediction computer programs, such as AlphaFold, have made it increasingly easier to predict protein structure, they are generally reliant on multiple sequence alignments, and this means that they are unable to accurately predict the impacts of point mutations.^9^, ^10^ However, alternate computational methods using neural networks, decision trees and machine learning specifically designed for mutation analysis are available.^11^ The ability to use these in combination to understand how mutations may lead to AMR will substantially improve rational structure‐based drug design of antibiotics. Moreover, predicting antibiotic resistance will enable drug developers to better select candidate drugs during early‐stage screening, while also potentially aiding in drug resistance surveillance. This could in turn change the cost–benefit scenario for many pharmaceutical companies and encourage them to restart development of antibiotics. In this study, we systematically analyze trends in drug‐resistant mutations in *emb* and *katG* and demonstrate the potential of using computational tool to predict drug resistance.

### 1.1 Ethambutol is a competitive inhibitor of *emb* complexes

**FIGURE 1:**
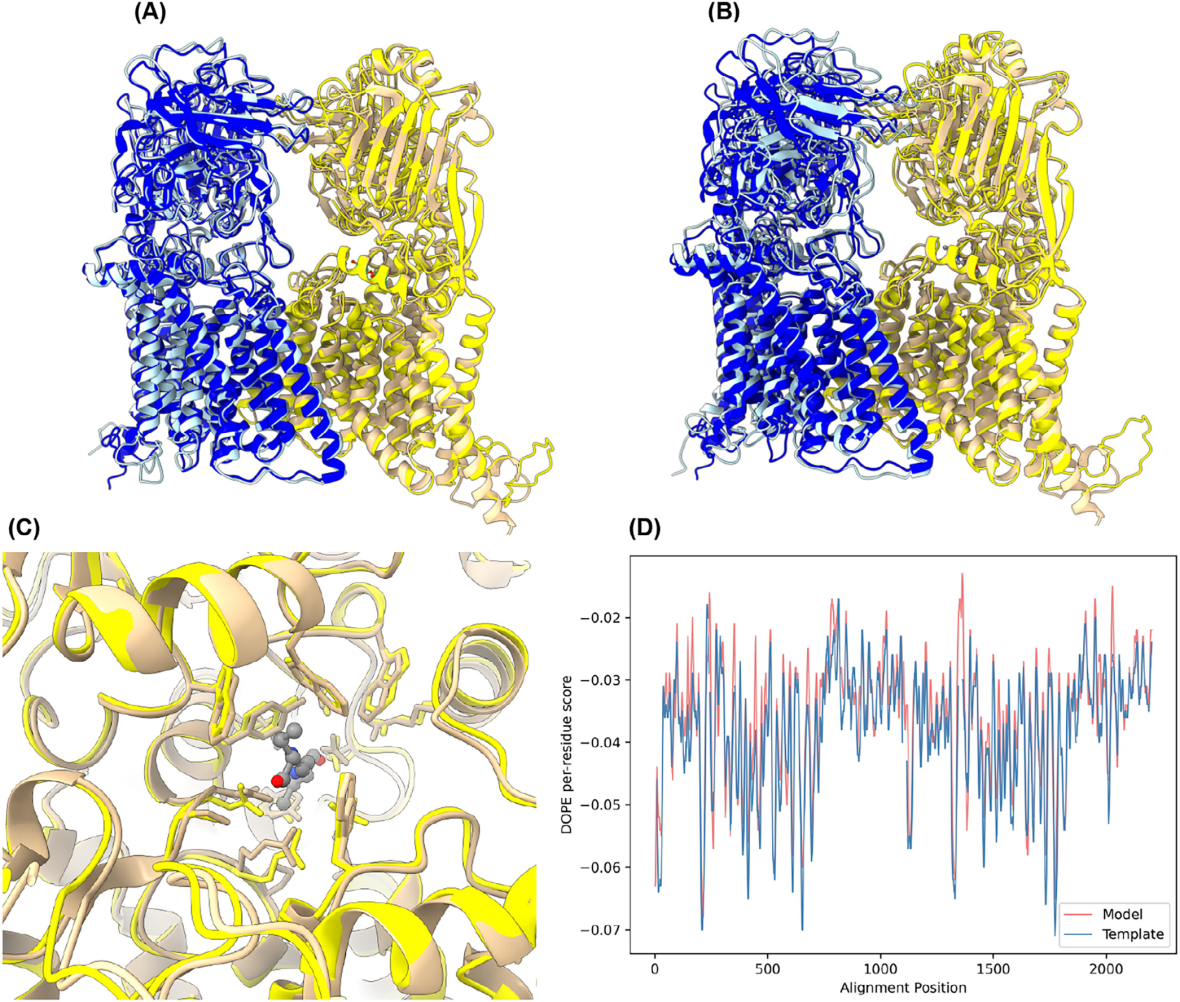
(A) Predicted structure of *embA‐embB*‐disaccharide complex superimposed with AlphaFold structures (P9WNL9, P9WNL7). On the left *embA* is shown with *embB* on the right (all atom RMSD for *embA* and *embB* across 8183 and 8331 atom pairs is 2.89 and 5.104 Å, respectively). (B) Predicted structure of *embA‐embB*‐ethambutol complex superimposed with AlphaFold structures. *embA* is on the left with *embB* on the right (all atom RMSD for *embA* and *embB* across 8183 and 8331 atoms pairs is 3.32 and 5.04 Å, respectively). (C) Active site of predicted *embB* structure with ethambutol superimposed with AlphaFold prediction showing good structural agreement in the side chain orientation between the two structures. (D) DOPE score of predicted *Mtb embA‐embB*‐ethambutol (red) structure compared to the template—M. smegmatis *embA‐embB*‐ethambutol (blue). Predicted models using MODELLER for *embA* and *embB* are shown in light‐blue and yellow respectively. AlphaFold predicted models for *embA* and *embB* are shown in blue and tan respectively. Ethambutol in (C) is shown as ball and stick model colored by atom type.

Two *emb* complexes exist, composed of three *emb* proteins, *embA*, *embB*, and *embC*. Of these, *embA* and *embB* form a heterodimer, while *embC* forms a symmetric homodimer. Both complexes exist as membrane bound structures with large extracellular domains (Figure 1 and Supplementary Figure 1) and play essential roles in the complex synthesis of *M. tuberculosis* myolyl‐arabinogalactan‐peptidoglycan cell wall.^12^, ^13^, ^14^ Both complexes have also been shown to be essential to *Mtb* growth through knockouts, though interestingly, may not be essential in the closely related *Mycobacterium smegmatis*.^15^, ^16^ Recently, the structures for the *embA‐embB* heterodimer and *embC* homodimer in complex with either ethambutol or its natural substrate, di‐arabinose, were defined using cryo‐EM in the closely related *M. smegmatis*, with an *Mtb embA‐embB* heterodimer in complex with ethambutol also being resolved.^12^ Here, all five structures were also found to purify with an additional acyl‐carrier protein, *AcpM*.

Ethambutol and di‐arabinose were found to have overlapping binding pockets furthering the notion that ethambutol is a competitive inhibitor of the *emb* complexes. The Zhang group were also able to demonstrate that drug resistance arises in the *emb* complexes when mutations affect the affinity of the drug more drastically than the affinity of the substrate using known drug‐resistant mutants.^12^ Here we show that this holds true for a subset of identified *embB* mutations using computational methods to assess binding affinities of ethambutol and di‐arabinose.

### 1.2 Isoniazid is converted to its active form by *katG*

*katG*, a homodimer, is a heme‐dependent catalase‐peroxidase that can function either as a catalase, as a peroxidase or as a peroxynitritase using hydrogen peroxidase and a heme cofactor.^17^, ^18^ Its function in *Mtb* seems to be in protection against reactive oxygen and nitrogen intermediates produced by phagocytes and in the hostile macrophage environment it colonizes. However, the natural substrate(s) for *katG* and their binding sites have yet to be determined and are likely to bind promiscuously around the heme group. The structure of *katG* has been resolved by x‐ray crystallography in the related *Synechococcus elongatus* (with isoniazid) and *Mtb*, and more recently using cryo‐EM (with isoniazid), with these groups finding three potential binding sites for isoniazid that are in agreement with those in *M. smegmatis* and *Mtb*.^18^, ^19^, ^20^ Moreover, both groups also suggest that promiscuous binding, both at these three sites as well as other potential sites remains possible. We show that it is possible to corroborate these results computationally and recapitulate the three binding sites using AutoDock and Glide docking program.

Isoniazid is a prodrug and must be activated by *katG* before it is able to inhibit its target, *inhA*. Here, isoniazid undergoes heme‐dependent oxidation to form an activated molecule which then forms an activated INH‐NAD adduct that competitively inhibits *inhA*. Munir's research indicated that drug‐resistant mutations destabilized the heme group using two mutant proteins.^18^ We were able to extend these findings to a database of drug‐resistant mutations in *katG* and identify destabilizing mutants that tend to lower heme binding affinity compared to isoniazid.

## 2 RESULTS

### 2.1 Modeling *emb* complexes agrees with AlphaFold predictions

To explore drug resistance of the *emb* complexes in *Mtb* accurately, we used existing structures of *M. smegmatis emb* complexes as templates and used MODELLER, a homology modeling package, to create predicted *Mtb* structures.^12^, ^21^, ^22^ This was possible due to a high sequence identity and similarity between *Mtb* and *M. smegmatis* proteins (Table 1). While it was initially attempted to model the entire complex including the *AcpM* dimer that copurified with these complexes in *M. smegmatis*, this disrupted the orientation of individual subunits, leading to a higher Root Mean Square Difference (RMSD) when compared to predicted structures created by AlphaFold.^10^ Thus, these structures were modeled without *AcpM*.

**TABLE 1:** Pairwise sequence alignment between *Mycobacterium tuberculosis* and *Mycobacterium smegmatis*. Sequence identity is representative of exact pairwise matches between the two proteins, while sequence similarity is representative of amino acid pairs of similar chemical properties. The sequence alignment was carried out using EMBL EMBOSS Needle tool.^23^

Protein	Sequence identity	Sequence similarity
*embA*	67.0%	78.3%
*embB*	66.8%	78.7%
*embC*	73.7%	83.0%

We created four models, an *embA‐embB* heterodimer and an *embC*_*2*_ homodimer in complex with both ethambutol and disaccharide. These were found to have low all atom RMSD's when compared to predicted AlphaFold structures, with the backbone of the protein seemingly identical to the AlphaFold prediction (Figure 1A–C and Supplementary Figure 1A–C). The *embA‐embB*‐ethambutol complex was also modeled using Zhang's *M. smegmatis* structure, despite the group already resolving the *Mtb* structure, and this was done as a robustness check. Here, it was found that the two structures had a low RMSD (0.686 Å across 1010 atoms), further strengthening the validity of the models created. Finally, Discrete Optimized Protein Energy (DOPE) scores per residue were used to compare the energy with the original template and look for any large deviations, which would suggest a potential inaccuracy of the model (Figure 1D). Here, the vast majority of the protein follows closely with the original template, suggesting that our model has been created with high accuracy. One region of separation between DOPE scores, which can be seen approximately at residue 1300, shows the chain break between *embA* and *embB* and an N‐terminal insertion in *Mtb emb* that is not present in *M. smegmatis*. Thus, while this deviation between template and target DOPE scores is expected in this region, it is not likely to affect any conclusions reached on ethambutol binding. The DOPE score was mainly used to select the best model. Although the DOPE score profile for the model is consistently below zero (Figure 1D) as well as the model agreeing well with the predicted AlphaFold model (Figure 1C), caution must be exercised when analyzing resistance mutations that are distal to the drug binding site in order to account for potential allosteric effects. Similar results were also found for the *embC* homodimer (Supplementary Figure 1).

### 2.2 Molecular docking of isoniazid to *katG*

**FIGURE 2:**
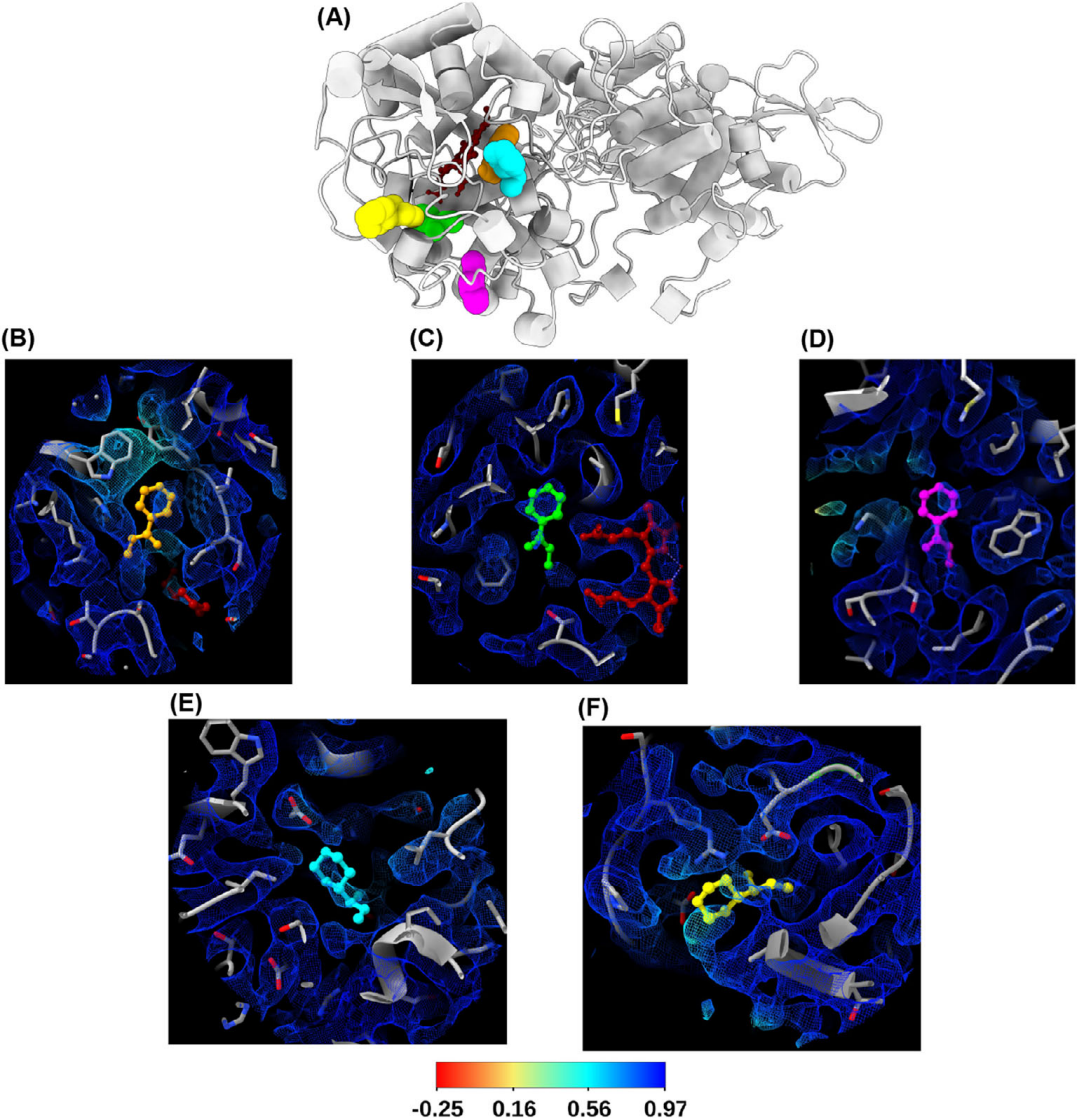
Predicted binding modes of isoniazid and its corresponding unresolved cryo‐EM densities identified by EMDA map‐model difference analysis. (A) Predicted binding modes of isoniazid identified at site 1 by AutoDock (orange) and Glide (cyan), site 2 identified by AutoDock (green) and Glide (yellow), and site 3 identified by AutoDock (magenta). (B–F) Binding modes shown in (A) with its corresponding unresolved cryo‐EM densities (EMDB 11776). Map‐model local correlation values calculated by EMDA are used to color the EM map surface (shown as mesh) in order to detect unresolved densities. Map regions with low correlation values represent potential isoniazid binding sites. Color gradient (shown as color key at the bottom) from red to blue corresponds to low and high correlation values respectively.

Despite *katG* structures being studied extensively, both using x‐ray crystallography and cryo‐EM, the binding site for isoniazid has still not been conclusively found in *M. tuberculosis*, perhaps due to the possibility of promiscuous binding.^18^, ^19^, ^20^ Using two independent docking programs (AutoDock^24^ and Glide^25^), we validate Munir et al.'s results, which showed cryo‐EM densities currently unresolved but most likely attributable to isoniazid in *M. tuberculosis* (EMDB 11776,^18^) (Table 2). Here, we focus on all three sites (sites 1–3) identified by Munir et al. that are located around the heme group and therefore most likely to be catalytically active. Here we use EMDA^26^ to the detect differences between map and the model and identified unresolved cryo‐EM densities that overlap with results from docking (Figure 2).^18^

**TABLE 2:** AutoDock and Glide predicted binding modes and affinities for sites 1–3 obtained by docking isoniazid onto *katG*. AutoDock was used to sample the entire protein (identifying binding mode at site 2) and the region proximal to the heme group (identifying binding modes at sites 1 and 3). Glide docking identified additional binding modes in sites 1 and 2.

Site ID	AutoDock Rank (score in kcal/mol)	Glide Rank (score in kcal/mol)
Site 1	Rank 3 (−5.2)	Rank 1 (−7.0)
Site 2	Rank 8 (−5.5)	Rank 9 (−6.3)
Site 3	Rank 1 (−5.4)	‐

This, alongside complementary evidence in *S. elongatus* and hotspot analysis, strengthens the argument that these are biologically relevant binding sites, although it does not rule out the possibility of additional binding conformations or sites. Moreover, the results of docking in combination with cryo‐EM map‐model difference analysis suggest multiple conformations at site 1, 2, and 3 further promoting the concept that isoniazid may bind promiscuously to *katG* at multiple binding sites (Table 2, Figure 2).

### 2.3 Analyses of the effects of drug resistance mutations on the protein stability

**FIGURE 3:**
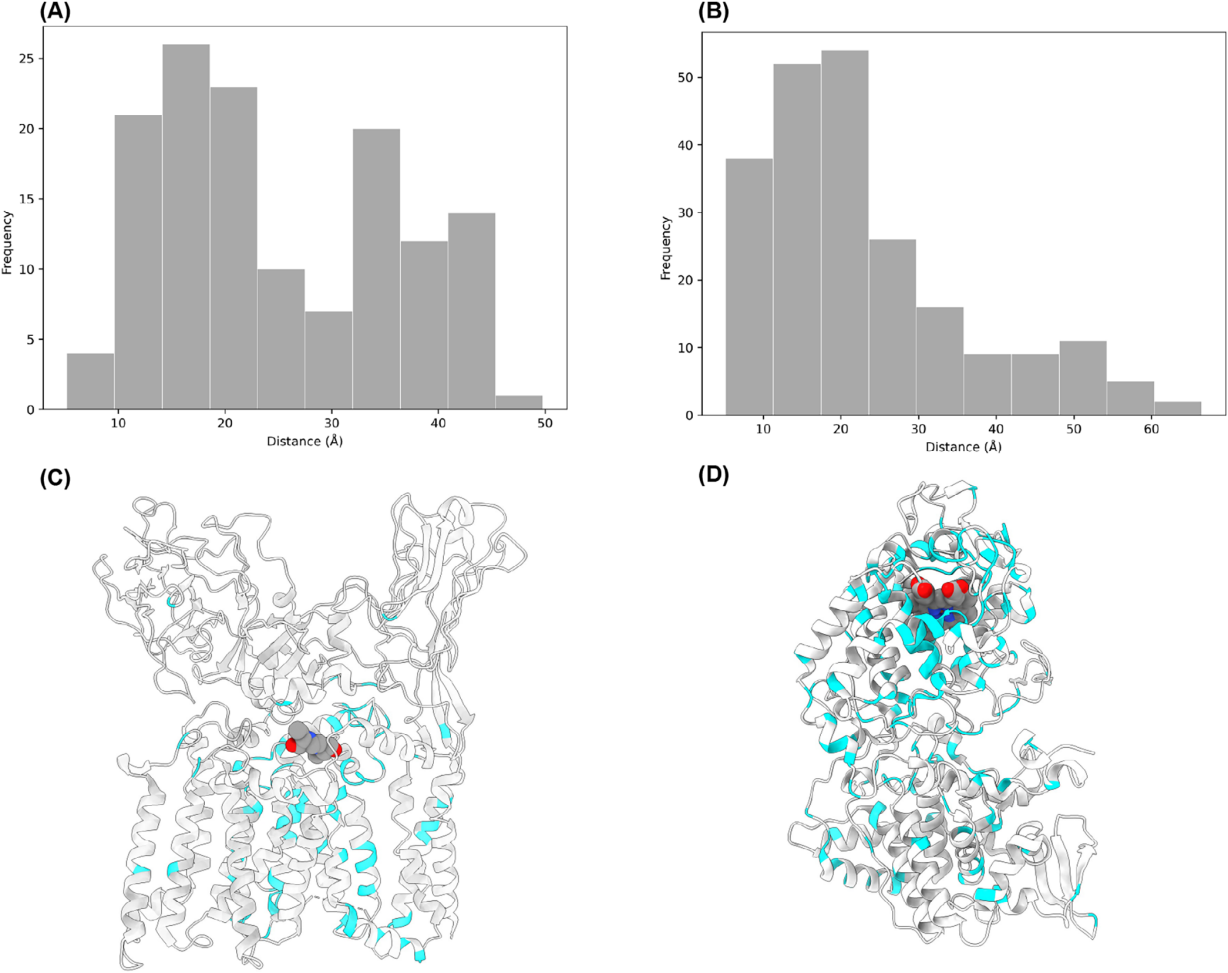
Drug‐resistance mutations in *embB* and *katG*. (A) Histogram showing the bimodal distribution of distances (Å) from the active site that drug‐resistant mutations occur in *embB*. (B) Histogram showing the distribution of distances (Å) from the active site that drug‐resistant mutations occur in *katG*. (C) *embB* (gray) with drug‐resistant mutations (cyan) and ethambutol (sphere model colored by atom type). (D) *katG* (gray) with drug‐resistant mutations (cyan) and heme (sphere model colored by atoms type).

To evaluate the impacts of drug‐resistant mutations on the structure of the protein, we studied the typical characteristics of the mutations that lead to drug resistance. We analyzed the distances of these mutations from the active site for *embB* and *katG* (Figure 3). This showed several mutations occurring close to the active site for both *embB* and *katG*. Moreover, in the case of *embB*, there is a bimodal distribution, and an additional peak of mutations that occur approximately 40 Å from the active site. This is likely attributable to the occurrence of these mutations in the membrane domain of *embB* (Figure 3A,C).

**FIGURE 4:**
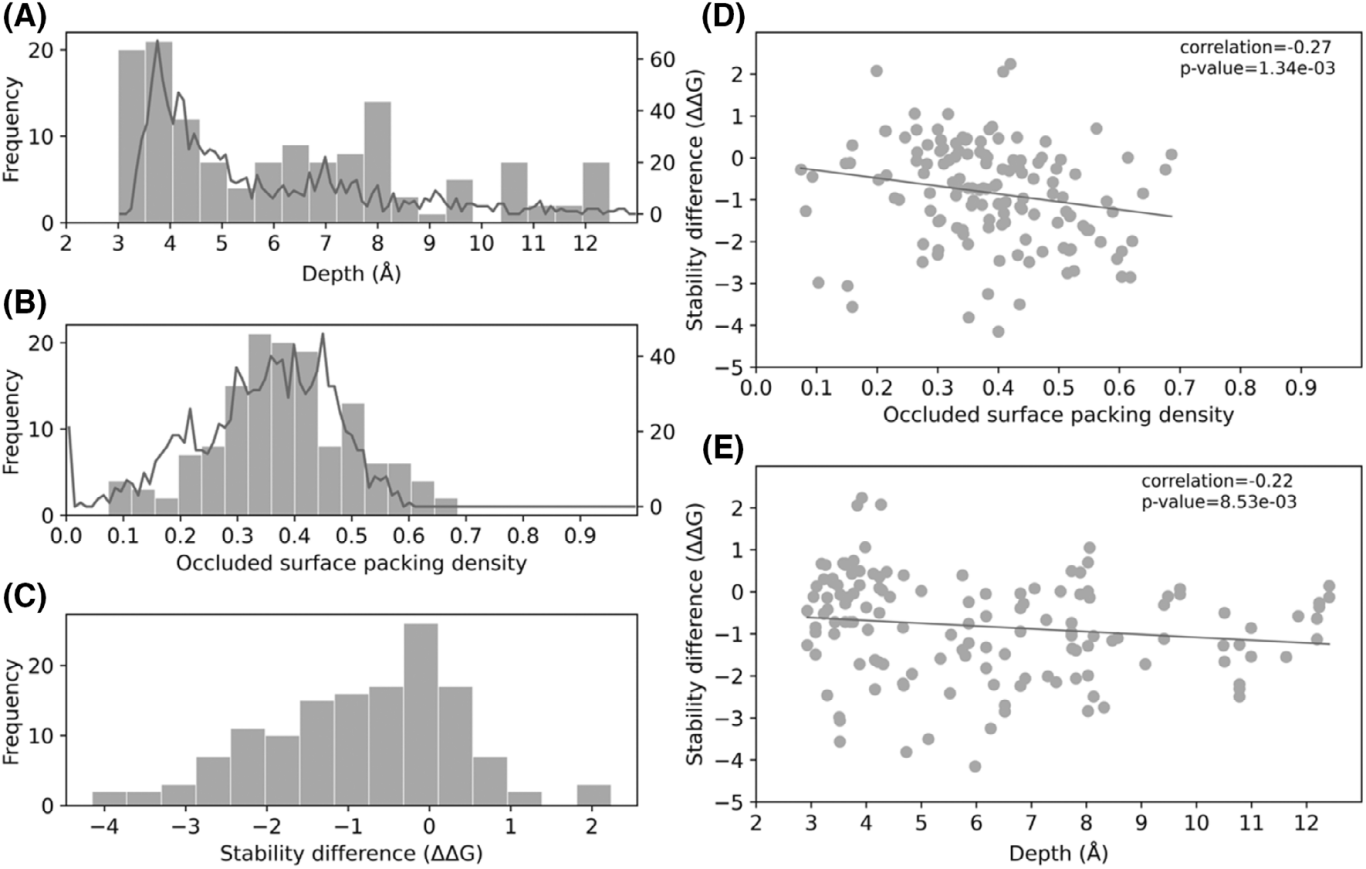
SDM mutant stability prediction and the structural properties of drug‐resistance mutations in *embB*. (A) Histogram showing distribution of residue depth (Å) of drug‐resistant mutations and line graph of distribution of depth (Å) of all residues in *embB*. The residue depth distribution of drug‐resistant mutations follows the distribution of residue depth across the entire protein except at 3 and 8 Å. (B) Histogram showing the distribution of occluded surface packing density (OSP) of drug‐resistant mutations in *embB* and line graph showing the distribution of OSP for all residues. The distribution of drug‐resistant mutations follows the distribution of residue OSP. (C) Histogram showing the distribution of change in stability (ΔΔG) caused by drug‐resistant mutations in *embB*, calculated by SDM. (D) Scatter plot of SDM stability difference (ΔΔG) of drug‐resistant mutations in *embB* against OSP with ordinary least squares regression (Spearman correlation *R* = −0.27, *p*‐value = 1.34e⁻^03^). (E) Scatter plot of SDM stability difference (ΔΔG) of drug‐resistant mutations in *embB* against residue depth (Å) with ordinary least squares regression (Spearman correlation: *R* = −0.22, *p*‐value = 8.53e⁻^03^).

**FIGURE 5:**
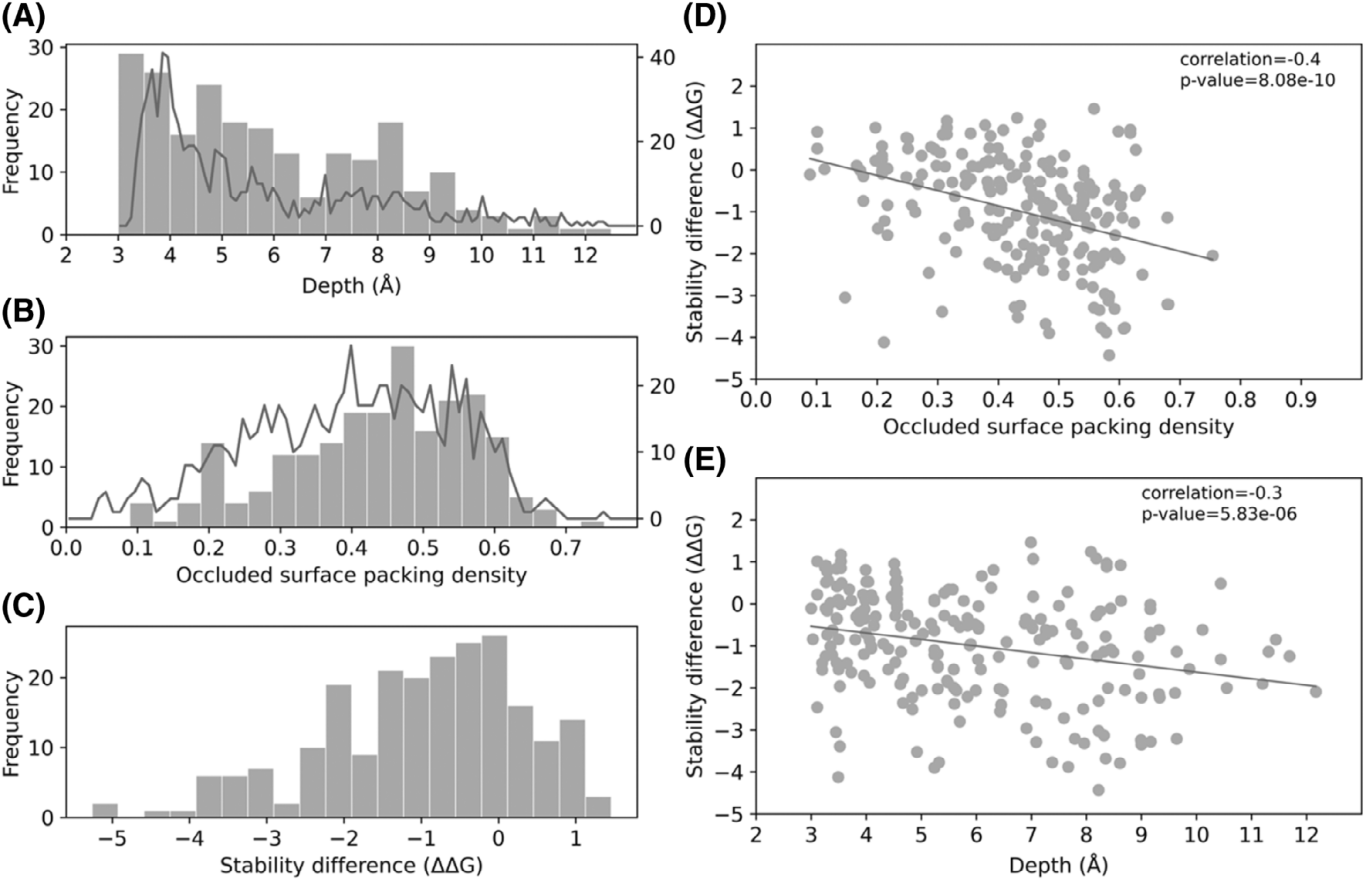
SDM mutant stability prediction and the structural properties of drug‐resistance mutations in *katG*. (A–E) same as Figure 4 but for *katG*. (A) Histogram showing distribution of residue depth (Å) of drug‐resistant mutations and line graph of distribution of depth (Å) of all residues in *katG*. The distribution of drug‐resistant mutations follows the distribution of depth across the entire protein except at 3 and 8 Å. (B) Histogram showing the distribution of occluded surface packing density (OSP) of drug‐resistant mutations in *katG* and line graph showing the distribution of OSP for all residues. The distribution of drug‐resistant mutations seems to be skewed toward higher OSP values. (C) Histogram showing the distribution of change in stability (ΔΔG) caused by drug‐resistant mutations in *katG* calculated by SDM. (D) Scatter plot of stability difference (ΔΔG) of drug‐resistant mutations in *katG* against occluded surface packing (OSP) with ordinary least squares regression (Spearman correlation *R* = −0.4, *p*‐value = 8.08e⁻^10^). (E) Scatter plot of stability difference (ΔΔG) of drug‐resistant mutations in *katG* against residue depth (Å) with ordinary least squares regression (Spearman correlation: *R* = −0.3, *p*‐value = 5.83e⁻^06^).

To further evaluate the characteristics of resistant mutations, we looked at the distribution of residue depth and Occluded Surface Packing density (OSP), and calculate the change in stability due to resistant mutations for *embB* (Figure 4) and *katG* (Figure 5) using site directed mutator program (SDM).^27^, ^28^ Similar analysis was also carried out for *embC* and shown to be overall consistent with results found for *embB* (Supplementary Figure 2). Results for *embA* are not shown due to limited availability of data. The distribution of residue depth at which drug‐resistant mutations occur is approximately equal to the distribution of depth of the entire protein except at low depths (3 Å) and at more buried depths (8 Å) for both *embB* and *katG* (Figures 4A and 5A). The distribution of residue OSP for drug‐resistant mutations is approximately equal to the distribution of OSP of the entire protein in *embB*, while for *katG* drug‐resistant mutations tend to have a higher OSP (Figures 4B and 5B). Finally, looking at the stability difference, one can see that in both *embB* (*n* = 138, *x¯*= − 0.832) and in *katG* (*n* = 222, *x¯*= − 0.992) there is a slight tendency toward destabilizing mutations. Carrying out a t‐test, we show that the stability differences in *katG* and *embB* are not statistically different. Next, we confirmed that there is a correlation between difference in stability and OSP or depth (Figures 4D,E, 5D,E). Correlation with both depth and OSP is lower in *embB*, though this is not unexpected given that *embB* is a membrane protein. Previously, we have shown that both the OSP and residue depth could act as an important parameter to understand the impact of mutations on protein stability and interaction.^29^

**FIGURE 6:**
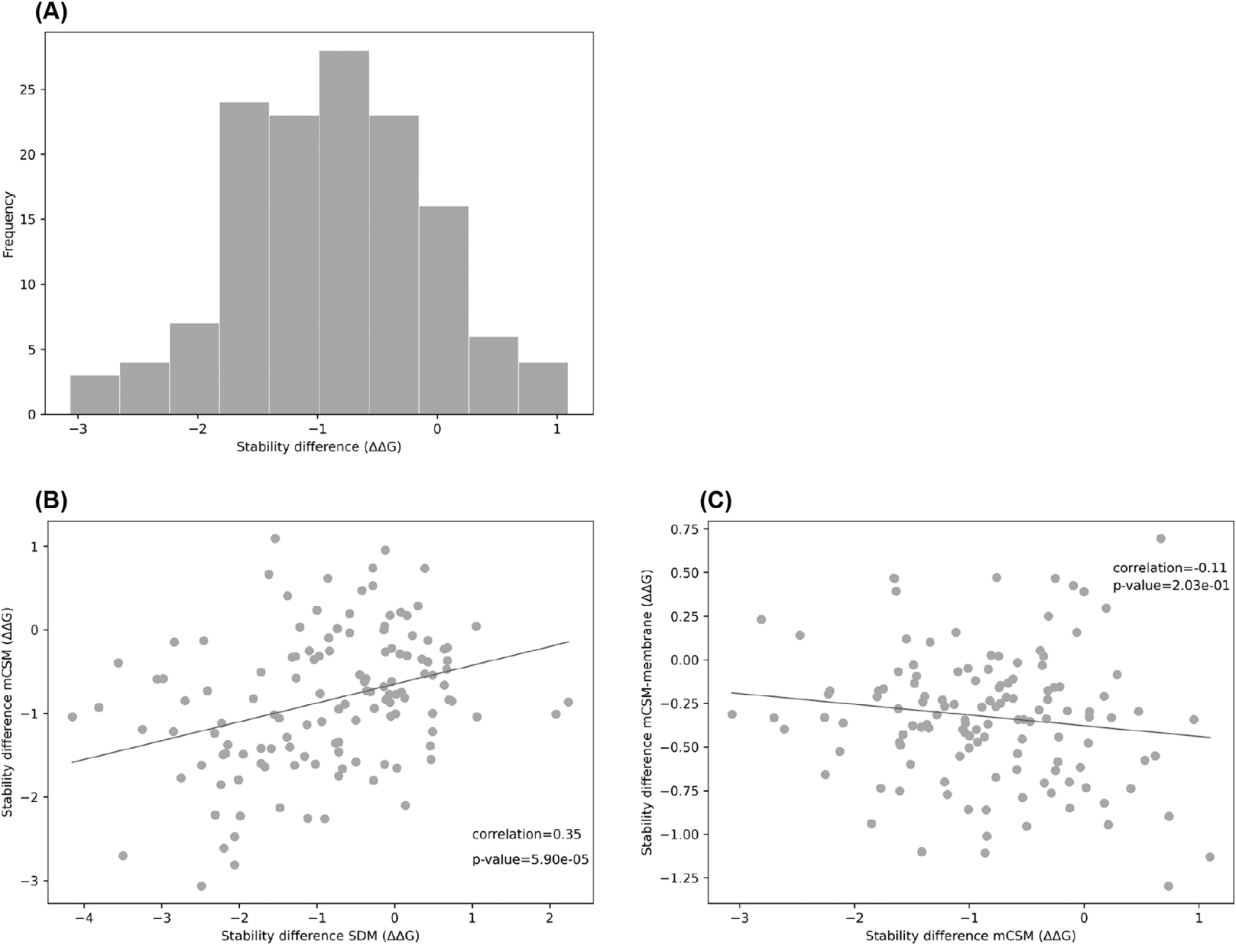
(A) Histogram showing the distribution of change in stability (ΔΔG) caused by drug‐resistant mutations in *embB* calculated by mCSM. (B) Scatter plot of change in stability (ΔΔG) calculated by mCSM against change in stability (ΔΔG) calculated by SDM for drug‐resistant mutations in *embB*, with an OLS regression (Spearman correlation: *R* = 0.35, *p*‐value = 5.90e⁻^05^). (C) Scatter plot of change in stability (ΔΔG) calculated by mCSM‐membrane against change in stability (ΔΔG) calculated by mCSM for drug‐resistant mutations in *embB*, with an OLS regression (Spearman correlation: *R* = −0.11, p‐value = 2.03e⁻^01^).

**FIGURE 7:**
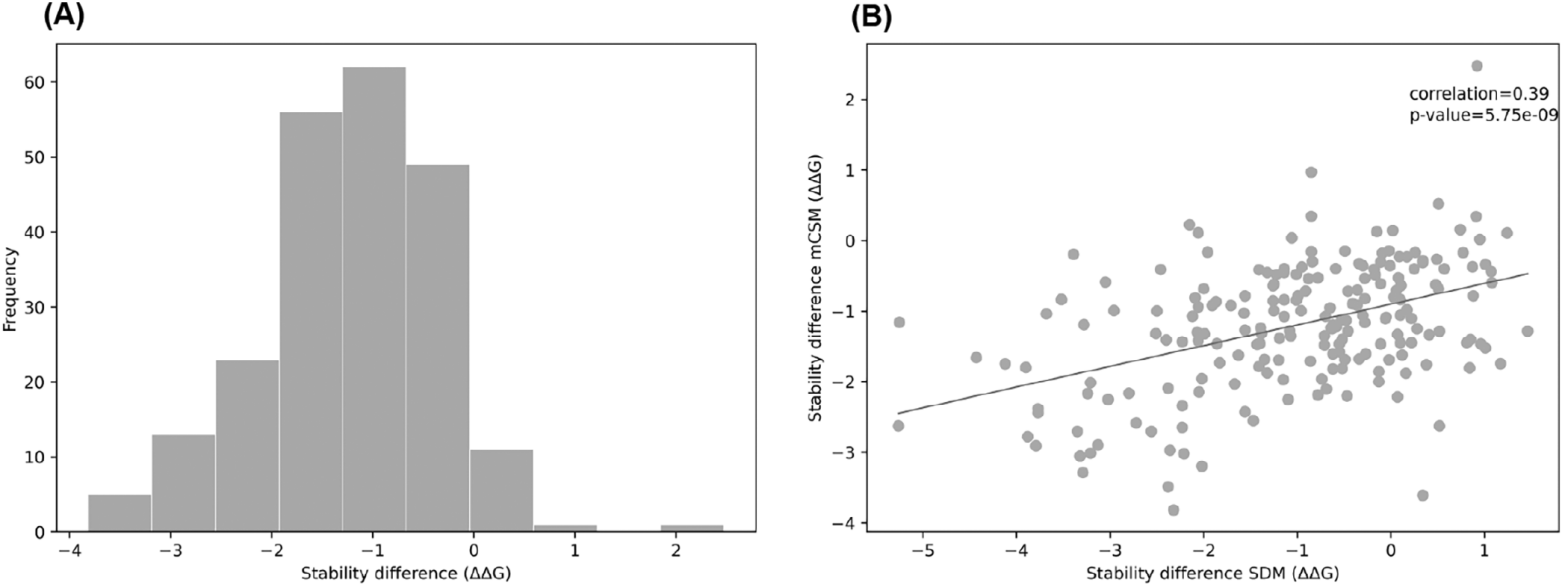
(A) Histogram showing the distribution of change in stability (ΔΔG) caused by drug‐resistant mutations in *katG* calculated by mCSM. (B) Scatter plot of change in stability (ΔΔG) calculated by mCSM against change in stability (ΔΔG) calculated by SDM for drug‐resistant mutations in *katG*, with an OLS regression (Spearman correlation: *R* = 0.39, *p*‐value = 5.75e⁻^09^).

Validation of results was then achieved by using mutation Cutoff Scanning Matrix (mCSM), a machine‐learning approach to calculate stability changes as opposed to the knowledge‐based approach used by SDM.^30^ The mCSM stability difference scores for *embB* are normally distributed (Figure 6A). The stability difference scores between SDM and mCSM are moderately correlated (Figure 6B). As *embB* is a known membrane protein, we also used mCSM‐membrane, an offshoot of mCSM created specifically for membrane proteins.^31^ In contrast to what is seen between SDM and mCSM, there is no correlation between stability scores computed by mCSM and mCSM‐membrane (Figure 6C). This highlights the fact that the substitution patterns and the mutant environment between soluble and membrane proteins are distinct, and an appropriate mutant stability predictor tailored for membrane proteins should be used. Similarly, for *katG*, the mCSM stability difference scores are normally distributed and the stability scores between SDM and mCSM are moderately correlated (Figure 7).

### 2.4 Impact of drug resistance mutations on binding affinity

**FIGURE 8:**
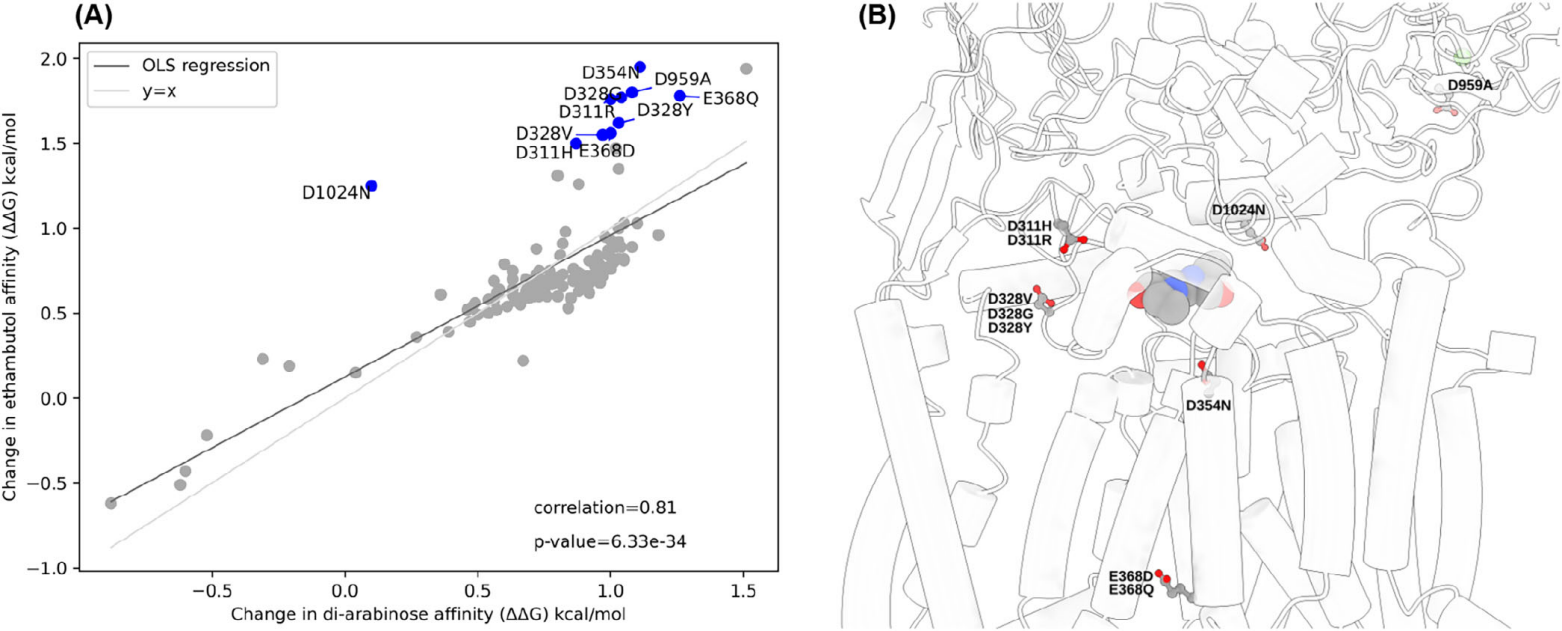
(A**)** Scatter plot of affinity change of ethambutol (ΔΔG) against affinity change of disaccharide (ΔΔG) with Ordinary Least Square (OLS) regression (Spearman correlation *R* = 0.81, *p*‐value = 6.33e⁻^34^) calculated by PremPLI. The line y = x is also plotted to show the theoretical line of best fit if both ethambutol and disaccharide affinities were affected equally. Drug resistance mutations (shown in blue) reducing ethambutol binding affinities relatively more compared to di‐arabinose were identified by finding points with the largest residuals after carrying out OLS regression. (B) Mutations shown in blue (A) are mapped on to *embB* bound to ethambutol (PDB 7BVF). *embB*, wildtype residue of the mutants and ethambutol are shown as cartoon, ball and stick and sphere models, respectively.

**FIGURE 9:**
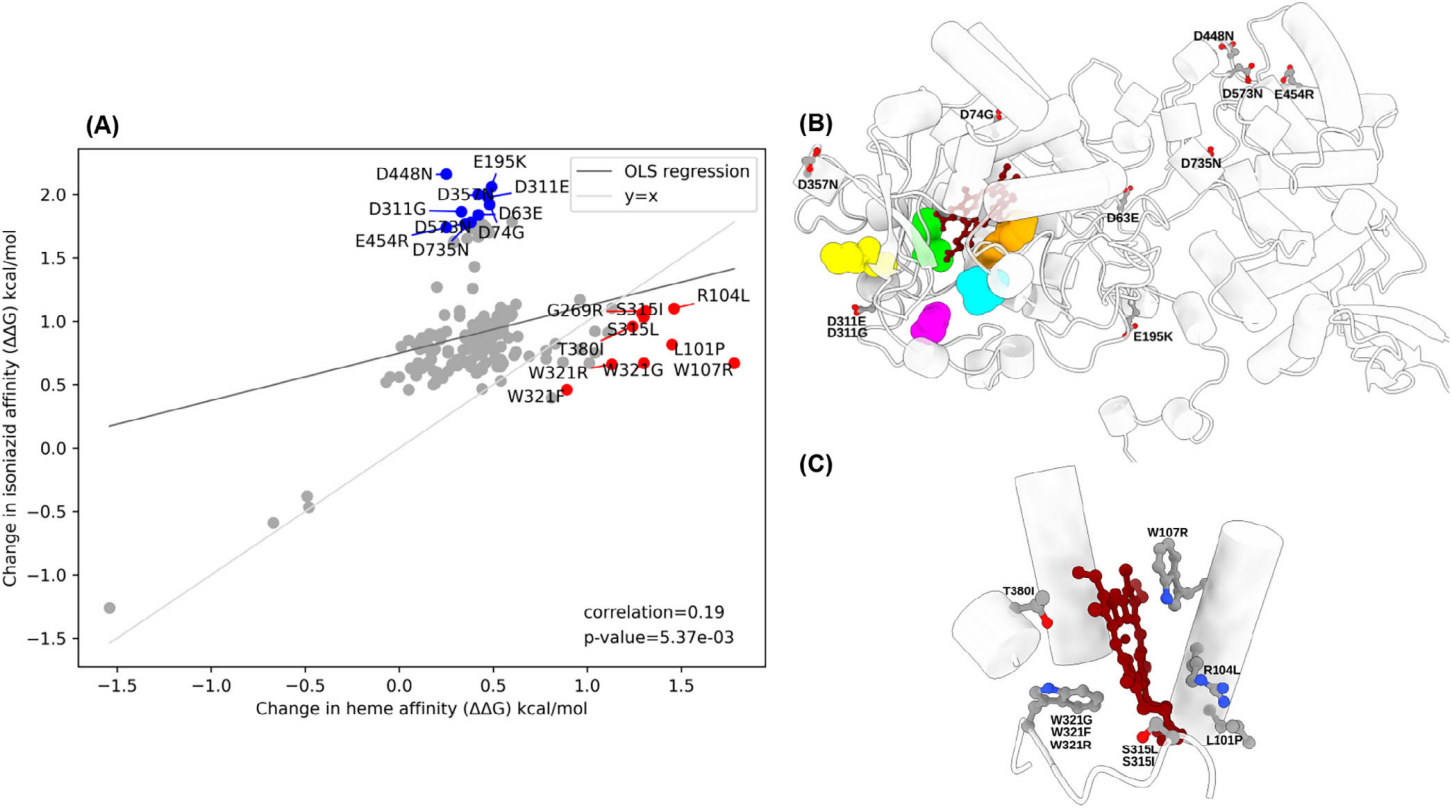
(A**)** Scatter plot of affinity change of isoniazid (ΔΔG) against affinity change of heme (ΔΔG) with Ordinary Least Square (OLS) regression (Spearman correlation *R* = 0.19, *p*‐value = 5.37e⁻^03^) calculated by PremPLI. The line y = x is also plotted to show the line of best fit when both isoniazid and heme affinities were affected equally. Drug resistance mutations reducing isoniazid and heme binding are shown in blue and red respectively. (B) Mutations identified in (A, in blue) are mapped on to *katG* (PDB 2CCA). Predicted binding modes of isoniazid identified at site 1 by AutoDock (orange) and Glide (cyan), site 2 identified by AutoDock (green) and Glide (yellow), and site 3 identified by AutoDock (magenta). (C) Mutations identified in (A, in red) are mapped on to *katG*. *katG* is shown as cartoon with wildtype residue of the mutants and heme are shown as ball and stick colored by atom type. Heme is shown in red.

To evaluate whether different mechanisms of acquiring drug resistance could be distinguished computationally, we used PremPLI, a software designed to compute the change in ligand affinity after the protein is mutated.^32^ Here, we attempted to recapitulate results which showed, for a limited number of mutations, that resistance in *embB* was achieved when the affinity of ethambutol was reduced. In the case of *katG*, we identified resistance mutations associated with heme destabilization^12^, ^18^ as well as identifying mutations that destabilize isoniazid binding relatively more compared to heme. We tested the affinity change to both ethambutol and di‐arabinose in *embA‐embB* heterodimer (Figure 8), and isoniazid and heme affinity in *katG* (Figure 9). It was found that the change in ethambutol and di‐arabinose affinities upon drug resistance mutations are highly correlated (*R* = 0.81). Based on the comparative analysis we identified 10 outlier mutations that preferentially affect ethambutol binding compared to di‐arabinose (Figure 8A). Interesting in all 10 cases, either an aspartate or a glutamate residue was involved in the mutation. Seven out of 10 mutations are found at the interface between the transmembrane and periplasmic domain of *embB* and within 10 to 20 Å distance from the ethambutol binding site (Figure 8B). These mutations either individually or in combination could affect the conformational flexibility of the interface that defines ethambutol binding thereby affecting its affinity. In the case of *katG*, the change in binding affinity of isoniazid upon drug resistance mutation was Boltzmann weighted over 5 binding modes identified by AutoDock and Glide docking program (Table 2 and Figure 2). Unlike *embB*, for *katG*, the change in isoniazid and heme affinities upon drug resistance mutations are weakly correlated (*R* = 0.19) (Figure 9A). This is not surprising as isoniazid is known to bind to *katG* promiscuously. Based on the comparative analysis we identified two groups of outlier mutations that preferentially reduce ethambutol binding (Figure 9A, in blue) and heme binding (Figure 9A, in red). Interestingly, like *embB*, all identified mutations reducing isoniazid binding affinity involve either the aspartate or glutamate residue. The mutants are spread throughout the *katG* structure in relation to the heme binding and isoniazid predicted binding sites further supporting the promiscuous nature of isoniazid binding to *katG* (Figure 9B). We identified 9 resistance mutations that reduce heme binding compared to isoniazid (Figure 9C). They are found within 5 to 10 Å distance from the heme binding site. Interestingly, the analysis also identified the mutation W107R that lowers of heme affinity without affecting the affinity of isoniazid, consistent with previous results.^18^

## 3 DISCUSSION

This study explores trends in drug‐resistance in *M. tuberculosis*, specifically looking at two structurally and functionally distinct proteins implicated in the action of first‐line drugs ethambutol and isoniazid. A computational analysis has enabled the study of drug resistance in these proteins looking at a more exhaustive list of mutations curated from the literature, thus enabling more conclusive results on both how and why drug‐resistance arises in these proteins.^7^

Computational corroboration of results previously showing isoniazid binding at sites 1–3 (Table 2, Figure 2) reaffirms the notion of promiscuous binding.^18^ This is significant because an understanding of isoniazid binding will enable us to better understand how drug‐resistant mutations prevent the catalysis of isoniazid to its active form and enables rational design of isoniazid derivatives that could circumvent resistance.^33^ However, it is difficult to differentiate between biologically relevant promiscuous binding and the unavoidable noise that comes with methods such as docking, especially given that the predicted affinities are incredibly similar (Table 2). To address this challenge, we used two widely used independent docking programs (AutoDock and Glide) in combinations with map‐model difference analysis using cryo‐EM maps to validate predicted binding modes of isoniazid. This hybrid approach allows a better understanding of the relationship between drug binding and resistance mutations.

As such, it is difficult to conclusively confirm that sites 1–3 are biologically relevant. however, considering the wealth of complementary evidence supporting this, it is tempting to suggest that they are. The next steps are to confirm which site(s) is responsible for the conversion of isoniazid to its active form. This is necessary to test potentially contradictory hypotheses that other binding sites could be present to simply increase the local concentration; or conversely, that any site proximal to heme is sufficient for catalysis; perhaps experiments such as NMR or surface plasma resonance could be exploited.^18^, ^19^, ^34^

It is evident from the analysis of the characteristics of drug‐resistant mutations in *embB* and *katG* that there are inherent similarities and differences in how drug resistance arises in *embB* and *katG*. In both, we see that several resistant mutations are centered near the active site (Figure 3A,B). This is largely expected for *katG*, as we would expect mutations that affect the active site to inhibit the *katG* catalysis and thus lead to drug resistance. In contrast, there are fewer mutations surrounding the active site of *embB*, perhaps reflecting the fact that *embB* is an essential protein and mutations near the active site may inhibit di‐arabinose binding as well as ethambutol binding. It is predicted, therefore, that these resistant mutations near the active site of *embB* must result in an inability to bind ethambutol, but cannot prevent di‐arabinose binding—at least not to an extent that it completely inhibits *emb* activity—and this is supported by PremPLI results (Figure 8).^12^, ^15^, ^16^ A large proportion of mutations are present approximately 40 Å from the active site, most likely within the membrane domain (Figure 3A,C). It is thus tempting to speculate that there are perhaps allosteric sites within the membrane domain that affect the structure of the binding site such that the affinity for ethambutol, but not di‐arabinose, is lowered. However, this needs to be confirmed by approaches such as molecular dynamics, NMR or cryo‐EM.^35^

In contrast, a subset of identified mutations, occurring near the active site of *katG*, is likely to have the opposite effect and seem to preferentially destabilize the heme group rather than affecting isoniazid affinity at any binding site (Figure 9), a finding complementary to previous studies.^18^ Perhaps this can be considered further evidence that there are multiple isoniazid binding sites, given that resistance mutations affecting isoniazid affinity would then need to affect multiple binding sites, something that is unlikely to happen.

Interestingly, given that *katG* is hypothesized to protect *Mtb* against the phagocytic oxidative burst and the highly oxidative macrophage environment that it infects, it would be intriguing to study isoniazid‐resistant strains for increased susceptibility to ROS (Master et al., 2001; Ng et al., 2004). Indeed, it has been observed that knockout mutants of *katG* are impaired in the presence of host ROS.^36^ Given that *katG* mutants with a destabilized heme group are observed to have no catalase activity and contrasting effects on peroxidase activity, it is tempting to therefore speculate that *katG* mutants conferring isoniazid resistance would be more susceptible to ROS.^18^, ^37^, ^38^ This is especially true given that observations of increased peroxidase activity are hypothesized to result from an increased stabilization of an iron(IV)‐oxo containing intermediate, and given that we show heme destabilization this is unlikely to hold true for mutants resulting in isoniazid resistance, though this will need to be further investigated.^39^ Based on these results, it could therefore be possible to further target isoniazid‐resistant TB with ROS.

It is also interesting to note that studying the change in predicted binding affinity upon mutation can successfully differentiate between different types of drug resistance in *embB* and *katG*. This could have positive implications for our ability to create a tool for predicting potential drug resistance sites. Advances in next generation sequencing technologies and genome wide association studies coupled with the recent developments of machine learning approaches for accurate protein structure prediction will aid the development of novel methods to predict drug‐resistance.^10^, ^40^, ^41^, ^42^ Moreover, given that resistance mutants seem to occur at specific residue depths and distances, but do not tend to massively affect the stability of the protein, these could also be used as parameters in a predictive tool. However, the limited number of neutral mutants further challenge the ability to differentiate between resistant and neutral mutations. The lack of neutral mutations is most probably a result of the literature in general not including naturally occurring single nucleotide polymorphisms, although this could likely be found from genome‐wide studies carried out on TB. Nonetheless, a lack of data on neutral mutations will make it both difficult to test the potential distinguishing power of such a tool and to train any form of machine learning techniques.^43^

However, given the weak correlation between the SDM and mCSM, it is important that protein stability in the context of drug‐resistant mutations be further investigated using hybrid approaches in combination with force‐field‐based methods like FoldX.^44^ The reasons for weak correlation between SDM and mCSM could be for a variety of reasons, including the fact that computationally calculating protein stability changes has traditionally been a challenge, the fact that mCSM and SDM are based on two very different methodologies and the fact that *emb* is a membrane protein. However, it is important to note that SDM and mCSM have been used without any additional optimization of the input parameters and therefore there is a possibility that these results could be further improved. Nonetheless, these results currently indicate that there are significant challenges in predicting protein stability and highlights the need for the development of novel methods to understand protein stability in the context of drug‐resistant mutations.

## 4 METHODS

### 4.1 MODELLER

MODELLER is a homology or comparative protein structure modeling method.^21^ It can use a template with known structure aligned to the target sequence to create and satisfy spatial and physical restraints. It is for this reason that MODELLER works well on targets with high sequence similarity, as is the case with *emb* proteins. We first edited PDB files for *embA‐embB* and *embC*_*2*_ such that they did not contain *AcpM*_*2*_, for reasons discussed in the results section (PDB files 7BVC, 7BVG, 7BVE, 7BVH^12^). We next created a sequence alignment between our known *M. smegmatis* structures, and the *M. tuberculosis* sequences, treating all ligands present in the PDB files as rigid bodies. This was thought to be a reasonable assumption given that ethambutol and disaccharide are relatively small and are expected to bind to the active site of *Mtb* in the same conformation as *M. smegmatis*. Using MODELLER's in‐built sequence alignment protocol has several benefits, namely that it can consider structural information from the template when creating an alignment, resulting in more accurate sequence alignments. Using this alignment, we created five models for each structure. We then selected the most accurate model using the lowest overall DOPE score.

### 4.2 AutoDock Vina and Glide

AutoDock Vina is a computational docking tool to predict noncovalent binding of macromolecules and ligands, alongside their binding affinities, by attempting to score the chemical potential and, from this, the free energy of binding and the affinity.^24^, ^45^ Here, we used Chimera to prepare isoniazid for docking with *katG* (PDB 2CCA) using *Vina*.^20^, ^46^ We ran two docking experiments, the first over the entire protein, and the second only proximal to the heme group, using default settings for energy range (3), exhaustiveness (8), and the number of binding modes generated (9). The purpose of using a high energy range was to look for binding modes of varying affinity throughout the protein, while the purpose of a high exhaustiveness was to increase the accuracy of the binding modes found by increasing the probability of finding the global minima. To address the promiscuous nature of isoniazid binding we additionally used Glide docking program to generate 100 docking solutions with default parameters.^25^ The dimension of the docking grid was set to 75 Å^3^ centered around the heme binding pocket. Ligand flexibility is allowed both in AutoDock and Glide docking.

### 4.3 EMDA difference map analysis

Previously, the cryo‐EM structure of *katG* dimer was resolved at 2.7 Å in the presence of isoniazid (EMDB 11776) suggesting three potential binding sites (sites 1–3) around the heme binding pocket.^18^ But it was not possible to resolve the binding modes of isoniazid due to the lack of atomic resolution data. Here, we used electron microscopy data analysis (EMDA) program^26^ to compute a real space local correlation between map (EMDB 11776) and its corresponding fitted model (PDB 7AG8). The computed correlation map is used to color the EM map of *katG* in complex with isoniazid using ChimeraX.^47^ Regions of the map with low correlations values potentially corresponds to the unmodelled densities of isoniazid. The choice of color gradient is set from red to blue to correspond to low and high correlation values, respectively.

### 4.4 Preparation of data

To analyze the effect of drug resistance in *M. tuberculosis* we used the DRAGdb database, a manually created database for drug‐resistance in *Mtb*, containing mutations for *embA* (*n* = 32), *embB* (*n* = 206), *embC* (*n* = 35), and *katG* (*n* = 532).^7^ This database was cleaned such that it contained only single point mutations and mutations correctly aligned with the known *Mtb* sequence, resulting in the final dataset containing *n* = 11, 138, 31, 222 for *embA*, *embB*, *embC*, and *katG*, respectively. The datasets were also reformatted to fit the required format for SDM, mCSM, PremPLI, and mCSM‐membrane. The list of curated mutations is provided in the Supplementary Tables [Link], [Link].

### 4.5 Creating distance plots

BioPython was used to calculate the centroid of the active site.^48^, ^49^, ^50^ This allowed us to calculate the distance between the centroid of the active site and mutated amino acid residues.

### 4.6 SDM and mCSM

SDM (https://veena.medschl.cam.ac.uk/sdm2) is a knowledge‐based predictor of protein stability, based on conformationally constrained, environment‐specific substitution tables (ESSTs) derived from amino acid substitutions that are tolerated within protein families.^27^, ^28^ Here, we used our inhouse standalone version of SDM. In contrast, mCSM (https://biosig.lab.uq.edu.au/mcsm/stability) is a supervised machine learning algorithm trained on thermodynamic datasets which uses a graph‐based distance pattern based on atoms up to 30 Å away to consider environmental features.^30^ It is therefore a methodology distinct from SDM and was chosen to ensure that different methodologies did not lead to vastly different results. Here, we used the online version of mCSM and were therefore confined to default settings.

### 4.7 PremPLI

PremPLI (https://lilab.jysw.suda.edu.cn/research/PremPLI/) is a machine method to predict the change in ligand binding affinity upon on point mutations.^32^ It uses random forest regression scoring function to train on experimental data of binding affinity changes.

## AUTHOR CONTRIBUTIONS

**Aadam Basrai:** Writing – original draft; validation; investigation; data curation; visualization; writing – review and editing. **Tom Blundell:** Writing – review and editing; investigation; funding acquisition; supervision. **Arun Prasad Pandurangan:** Conceptualization; investigation; writing – original draft; writing – review and editing; supervision; methodology; data curation; validation; visualization; software.

## CONFLICT OF INTEREST STATEMENT

The authors declare no conflicts of interest.

### PEER REVIEW

The peer review history for this article is available at https://www.webofscience.com/api/gateway/wos/peer-review/10.1002/prot.26684.

## Supporting information


**Supplementary Figure 1.** (A) Predicted structure of *embC* homodimer disaccharide complex superimposed with AlphaFold structure (P9WNL5). The all‐atom RMSD for *embC* across 8302 atom pairs is 9.28 Å. (B) Predicted structure of *embC* homodimer ethambutol complex superimposed with AlphaFold structure. The all‐atom RMSD for *embC* is 7.32 Å. (C) Active site of predicted *embC* monomer structure with ethambutol superimposed with AlphaFold prediction showing good structural agreement in the side chain orientation between the two structures. (D) DOPE score of predicted *Mtb embC* homodimer with ethambutol structure (red) compared to the template—*M. smegmatis embC* homodimer with ethambutol (blue). Predicted models using MODELLER and AlphaFold for *embC* is shown in yellow and tan respectively. Ethambutol in (C) is shown as ball and stick model colored by atom type.
**Supplementary Figure 2.** SDM mutant stability prediction and the structural properties of drug resistance mutations in *embC*. (A) Histogram showing distribution of depth (Å) of drug‐resistant mutations and line graph of distribution of depth (Å) of all residues in *embC*. The residue depth distribution of drug‐resistant mutations follows the distribution of residue depth across the entire protein except around 3, 6, and 7 Å. (B) Histogram showing the distribution of occluded surface packing (OSP) of drug resistant mutations in *embC* and line graph showing the distribution of OSP for all residues. The distribution of drug‐resistant mutations follows the distribution of residue OSP except at high packing density values. (C) Histogram showing the distribution of change in stability (ΔΔG) caused by drug resistant mutations in *embC*, calculated by SDM. (D) Scatter plot of SDM stability difference (ΔΔG) of drug resistant mutations in *embC* against OSP with ordinary least squares regression (Spearman correlation *R* = −0.57, *p*‐value = 7.87e⁻^04^). (E) Scatter plot of SDM stability difference (ΔΔG) of drug resistant mutations in *embC* against residue depth (Å) with ordinary least squares regression (Spearman correlation: *R* = −0.57, *p*‐value = 8.04e⁻^04^).

**Supplementary Table 1.** Curated list of *embA* mutations.

**Supplementary Table 2.** Curated list of *embB* mutations.

**Supplementary Table 3.** Curated list of *embC* mutations.

**Supplementary Table 4.** Curated list of *katG* mutations.

## Data Availability

The data that supports the findings of this study are available in the supplementary material of this article.
