# Supplementary material for: Computational analyses of drug resistance mutations in katG and emb complexes in Mycobacterium tuberculosis
Source: Proteins. 2024 Mar 14;93(1):359–71. doi: 10.1002/prot.26684 (PMC11623437; doi:10.1002/prot.26684)
Supplement: Supplementary file 1 — Supplementary Figure 1. (A) Predicted structure of embC homodimer disaccharide complex superimposed with AlphaFold structure (P9WNL5). The all‐atom RMSD for embC across 8302 atom pairs is 9.28 Å. (B) Predicted structure of embC homodimer ethambutol complex superimposed with AlphaFold structure. The all‐atom RMSD for embC is 7.32 Å. (C) Active site of predicted embC monomer structure with ethambutol superimposed with AlphaFold prediction showing good structural agreement in the side chain orientation between the two structures. (D) DOPE score of predicted Mtb embC homodimer with ethambutol structure (red) compared to the template—M. smegmatis embC homodimer with ethambutol (blue). Predicted models using MODELLER and AlphaFold for embC is shown in yellow and tan respectively. Ethambutol in (C) is shown as ball and stick model colored by atom type. Supplementary Figure 2. SDM mutant stability prediction and the structural properties of drug resistance mutations in embC. (A) Histogram showing distribution of depth (Å) of drug‐resistant mutations and line graph of distribution of depth (Å) of all residues in embC. The residue depth distribution of drug‐resistant mutations follows the distribution of residue depth across the entire protein except around 3, 6, and 7 Å. (B) Histogram showing the distribution of occluded surface packing (OSP) of drug resistant mutations in embC and line graph showing the distribution of OSP for all residues. The distribution of drug‐resistant mutations follows the distribution of residue OSP except at high packing density values. (C) Histogram showing the distribution of change in stability (ΔΔG) caused by drug resistant mutations in embC, calculated by SDM. (D) Scatter plot of SDM stability difference (ΔΔG) of drug resistant mutations in embC against OSP with ordinary least squares regression (Spearman correlation R = −0.57, p‐value = 7.87e⁻04). (E) Scatter plot of SDM stability difference (ΔΔG) of drug resistant mutations in embC against r [file PROT-93-359-s002.docx]

**Supplementary Figure 1.** (A) Predicted structure of *embC* homodimer disaccharide complex superimposed with AlphaFold structure (P9WNL5). The all-atom RMSD for *embC* across 8302 atom pairs is 9.28 Å). (B) Predicted structure of *embC* homodimer ethambutol complex superimposed with AlphaFold structure. The all-atom RMSD for *embC* is 7.32 Å). (C) Active site of predicted *embC* monomer structure with ethambutol superimposed with AlphaFold prediction showing good structural agreement in the side chain orientation between the two structures. (D) DOPE score of predicted *Mtb* *embC* homodimer with ethambutol structure (red) compared to the template – M. smegmatis *embC* homodimer with ethambutol (blue). Predicted models using MODELLER and AlphaFold for *embC* is shown in yellow and tan respectively. Ethambutol in (C) is shown as ball and stick model coloured by atom type.


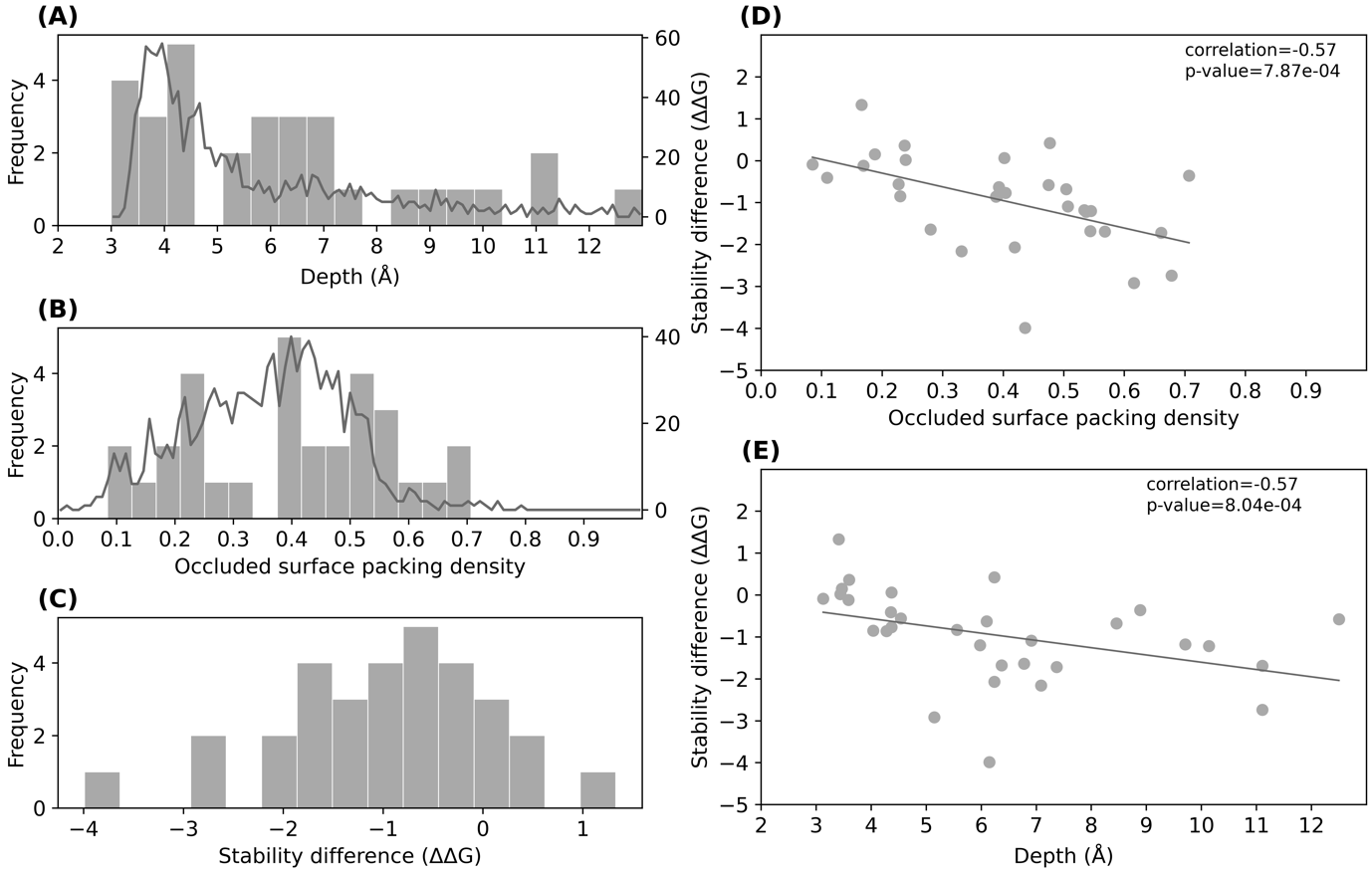


**Supplementary Figure 2.** SDM mutant stability prediction and the structural properties of drug resistance mutations in *embC*. (A) Histogram showing distribution of depth (Å) of drug-resistant mutations and line graph of distribution of depth (Å) of all residues in *embC*. The residue depth distribution of drug-resistant mutations follows the distribution of residue depth across the entire protein except around 3 Å, 6 Å and 7 Å. (B) Histogram showing the distribution of occluded surface packing (OSP) of drug resistant mutations in *embC* and line graph showing the distribution of OSP for all residues. The distribution of drug-resistant mutations follows the distribution of residue OSP except at high packing density values. (C) Histogram showing the distribution of change in stability (ΔΔG) caused by drug resistant mutations in *embC*, calculated by SDM. (D) Scatter plot of SDM stability difference (ΔΔG) of drug resistant mutations in *embC* against OSP with ordinary least squares regression (Spearman correlation R=-0.57, p-value=7.87e-04). (E) Scatter plot of SDM stability difference (ΔΔG) of drug resistant mutations in *embC* against residue depth (Å) with ordinary least squares regression (Spearman correlation: R=-0.57, p-value=8.04e-04).
