# Supplementary material for: Computational analyses of drug resistance mutations in katG and emb complexes in Mycobacterium tuberculosis
Source: Proteins. 2024 Mar 14;93(1):359–71. doi: 10.1002/prot.26684 (PMC11623437; doi:10.1002/prot.26684)
Supplement: Supplementary file 2 — Supplementary Table 1. Curated list of embA mutations. [file PROT-93-359-s004.docx]

**Supplementary Table 1.** Curated list of embA mutations

| **Mutation** |
| --- |
| V18F |
| A331T |
| P913S |
| G350D |
| G321S |
| P769T |
| G554D |
| A462V |
| A201T |
| G200S |
| D833A |
