# Supplementary material for: Computational analyses of drug resistance mutations in katG and emb complexes in Mycobacterium tuberculosis
Source: Proteins. 2024 Mar 14;93(1):359–71. doi: 10.1002/prot.26684 (PMC11623437; doi:10.1002/prot.26684)
Supplement: Supplementary file 3 — Supplementary Table 2. Curated list of embB mutations. [file PROT-93-359-s003.docx]

**Supplementary Table 2.** Curated list of embB mutations

| **Mutation** |
| --- |
| T437A |
| L370R |
| S366P |
| S380D |
| G374V |
| D328V |
| D311R |
| A357S |
| A310R |
| D328G |
| V369A |
| V282L |
| Q497K |
| V50A |
| V436G |
| V377M |
| F287J |
| N318S |
| L239P |
| A379D |
| V377G |
| M1000R |
| S347I |
| G406J |
| S297L |
| V452L |
| N399D |
| D240H |
| P430L |
| H1002R |
| M306T |
| A630I |
| A271V |
| M306I |
| Y319D |
| N399H |
| A221G |
| R274P |
| R460J |
| P397T |
| D354N |
| P375A |
| G406S |
| G294S |
| W395R |
| A659T |
| A434G |
| N399T |
| S272I |
| V435G |
| L359I |
| Q497H |
| Y384N |
| T643I |
| G331R |
| A679T |
| E378A |
| R471P |
| E368Q |
| G314E |
| A388G |
| Y319J |
| E368D |
| R469P |
| Y315L |
| D1024N |
| A379T |
| A225S |
| R128G |
| A357V |
| Q497P |
| G745D |
| N400P |
| A281P |
| V360M |
| N296K |
| F330V |
| Y334H |
| P461S |
| W332L |
| M423I |
| S347T |
| G406D |
| S366L |
| A281L |
| D354A |
| G459A |
| V282G |
| S380R |
| F287V |
| G448V |
| G406A |
| V369L |
| D299E |
| S412P |
| Y319S |
| E378K |
| I293N |
| P446H |
| Q497R |
| F398Y |
| R460L |
| R367P |
| I465D |
| D959A |
| T642A |
| G331Y |
| D328Y |
| A356S |
| E368A |
| Y333H |
| S297A |
| V227G |
| G314A |
| M306L |
| W395J |
| W322J |
| A431T |
| G401S |
| D311H |
| P397Q |
| H312R |
| G406P |
| S272J |
| M306V |
| G358V |
| F398H |
| E405P |
| N318K |
| A356V |
| N400K |
| V309G |
| S317T |
| Y319N |
| P371R |
| A356F |
| F285L |
| N318H |
