# Supplementary material for: Computational analyses of drug resistance mutations in katG and emb complexes in Mycobacterium tuberculosis
Source: Proteins. 2024 Mar 14;93(1):359–71. doi: 10.1002/prot.26684 (PMC11623437; doi:10.1002/prot.26684)
Supplement: Supplementary file 4 — Supplementary Table 3. Curated list of embC mutations. [file PROT-93-359-s005.docx]

**Supplementary Table 3**. Curated list of embC mutations

| **Mutation** |
| --- |
| A244T |
| N394D |
| G288W |
| D329E |
| G308D |
| R738Q |
| R302G |
| M300R |
| Y296H |
| V287F |
| M310K |
| V303G |
| A307T |
| H285Y |
| I297T |
| A254G |
| Y296S |
| A247P |
| P150S |
| G325S |
| A5S |
| I297L |
| Y327N |
| Q725R |
| T270I |
| G288V |
| L251R |
| V981L |
| Y309N |
| G272S |
| W326R |
