# Supplementary material for: Computational analyses of drug resistance mutations in katG and emb complexes in Mycobacterium tuberculosis
Source: Proteins. 2024 Mar 14;93(1):359–71. doi: 10.1002/prot.26684 (PMC11623437; doi:10.1002/prot.26684)
Supplement: Supplementary file 5 — Supplementary Table 4. Curated list of katG mutations. [file PROT-93-359-s001.docx]

**Supplementary Table 4.** Curated list of katG mutations

| **Mutation** |
| --- |
| W91R |
| T394M |
| A139P |
| D189G |
| S315G |
| S140N |
| T275A |
| L336P |
| S140A |
| P375R |
| A541D |
| D311G |
| A110V |
| S383P |
| G269D |
| R128Q |
| W300G |
| D735N |
| W505R |
| A727D |
| W341S |
| N258S |
| K274R |
| P232S |
| P131T |
| W321G |
| R104Q |
| D74Y |
| D94A |
| A291P |
| S315R |
| T306P |
| A281V |
| W341G |
| R418Q |
| R632J |
| A109V |
| W328G |
| H270R |
| A162V |
| N133T |
| A574V |
| Y229F |
| S303L |
| Y155J |
| A424E |
| G32D |
| P131R |
| D387H |
| M126I |
| D448N |
| S315L |
| W107R |
| D419H |
| E195K |
| L101P |
| R515J |
| Y98J |
| I393N |
| Q224E |
| A65T |
| G299J |
| T275S |
| D735A |
| Q88R |
| G491J |
| A550D |
| K488N |
| G299S |
| R489S |
| D357H |
| G629S |
| R515Y |
| L619P |
| R463G |
| T344P |
| Q127E |
| K414N |
| S315N |
| Y155S |
| A264T |
| R146W |
| Y337F |
| A162E |
| A350S |
| L634F |
| T667P |
| N238R |
| S315T |
| N138H |
| H108E |
| Y413J |
| A66P |
| H97R |
| A256T |
| R104L |
| M624V |
| P280S |
| G123E |
| W438S |
| R418L |
| G99E |
| M609I |
| K143T |
| T380I |
| D357N |
| S315I |
| Y304S |
| V47E |
| N529D |
| G451S |
| D573N |
| G118D |
| A106V |
| S303W |
| I71N |
| W328L |
| R128P |
| G234R |
| L141S |
| P131Q |
| L587P |
| P589T |
| F252L |
| G685R |
| D381G |
| N138T |
| Q295P |
| D194G |
| A350T |
| G169A |
| E454R |
| Q461P |
| V230A |
| W728J |
| V68G |
| D142A |
| G593D |
| D74G |
| G309D |
| K345T |
| I335T |
| Q127P |
| M105I |
| E217G |
| G279D |
| S457I |
| T394A |
| M176T |
| T262R |
| A243D |
| A61T |
| P429S |
| G316S |
| R498H |
| D387G |
| M84I |
| T275P |
| A243S |
| G316D |
| H270Q |
| I317L |
| A424V |
| G186V |
| A424G |
| N35D |
| F567S |
| H276M |
| P501A |
| M420T |
| D695A |
| D63E |
| S302R |
| M176I |
| N138D |
| Q295H |
| D142G |
| L148R |
| S160L |
| D194Y |
| A409D |
| H108Q |
| D542H |
| I335V |
| G494D |
| G285J |
| G212D |
| A172V |
| G269R |
| N596S |
| Y337J |
| G307E |
| R463L |
| G121D |
| T180J |
| E289D |
| L336R |
| V710A |
| V450D |
| N236T |
| A172T |
| W328J |
| T308P |
| N218K |
| W397Y |
| N138S |
| F658V |
| G485V |
| L587M |
| L148A |
| A93T |
| E318V |
| A409R |
| W321F |
| W321R |
| Q352E |
| G125J |
| S331J |
| R249J |
| R249H |
| W328S |
| D311E |
